# Refining restraint techniques for research pigs through habituation

**DOI:** 10.3389/fvets.2022.1016414

**Published:** 2022-09-23

**Authors:** Carly I. O'Malley, Raina Hubley, Halimatou Tambadou, Patricia V. Turner

**Affiliations:** ^1^Global Animal Welfare and Training, Charles River Laboratories, Wilmington, MA, United States; ^2^Department of Pathobiology, University of Guelph, Guelph, ON, Canada

**Keywords:** research animals, habituation, operant conditioning, pigs, restraint

## Abstract

Pigs are common research models and are strong animals that can be difficult to restrain. Improper restraint can put pigs and research personnel at risk for injury and induce stress, which can affect research outcomes. This study aimed to refine restraint techniques for research pigs using habituation and operant conditioning. Forty-four (22 males, 22 females; 4 months old, ~8.1 kg) Göttingen minipigs were randomly assigned to a control (C: no interventions) or a treatment group (T). Pigs in the T group received 3 min training sessions 3 days/week for the first 14 d after arrival. Training sessions included human socialization and habituation to a hammock sling for blood collection. Blood collection occurred on day 13 for all pigs by novel technicians. Pigs were placed in the sling, blood was collected from the radial vein, and serum cortisol levels were determined (ug/dL). Pig behavior was recorded and scored for duration of time spent struggling (s) and vocalizing (s). Novel human approach tests occurred on day 12, before blood collection, and day 14, after blood collection. Pigs were scored on latency to touch the human (s) and duration of time spent in contact with the human (s). Pig weight was taken upon arrival and on day 15. Separate linear models were fitted for response variables struggle duration in sling, serum cortisol, latency to touch human, time spent in contact with human, and body weight. Fixed effects were treatment and sex. Prior to blood collection, there was no difference in response to a novel human (*P* > 0.05) but after blood collection, T pigs were quicker to approach (estimate: −5.352, SE: 1.72, *P* = 0.003) and spent more time in contact with the novel human (estimate: 3.091, SE: 1.448, *P* = 0.039). T pigs also had lower cortisol levels during blood collection (estimate: −2.36, SE: 0.657, *P* = 0.001). There was no difference in behavior while in the sling (*P* > 0.05). The results of the study suggest that even small investments in habituation and training pigs to study procedures is beneficial in reducing stress and improving human-animal relationships, but more time would be beneficial to promote calmer behavior in the sling.

## Introduction

Pigs are useful biomedical models for studying human diseases because of similarities to humans in key traits such as anatomy, physiology, size, metabolic processes, and skin structure. They are used for a number of research applications including cardiovascular xenotransplantation, cancer, skin, and toxicology studies (1–3). However, pigs, including minipigs, are large, strong animals that can be difficult to restrain for study procedures. Improper restraint techniques for procedures such as blood collection can lead to stress and injury for both pigs and research personnel, which can result in altered biological parameters, invalidating research results (4–7). Previous work by Stephens and Rader found that pigs restrained in a harness with their feet lifted off the ground had increased heart rate and blood pressure and decreased renal blood flow compared to baseline measures from animals prior to restraint (4). Salivary proteins, body temperature, neurotransmitter expression (e.g., epinephrine, norepinephrine, dopamine), and cortisol levels can be altered in pigs due to stress from restraint (5–7).

One of the primary ethical principles when working with research animals is refinement, which aims to minimize pain and distress due to research procedures (8). Refinements not only serve as a means of improving animal welfare but are vital to ensuring high quality study data that is reproducible and translatable (9). Thus, refinement to restraint techniques for minipigs is warranted for both animal welfare and scientific purposes. The hammock sling is a refinement over manual restraint for minipigs as it is safer for both pigs and their handlers (10, 11). However, restraint in this type of sling, in which the limbs are also restrained, can still result in fear and discomfort in animals that are not habituated (12). Habituation is a form of non-associative learning in which response to a stimulus is decreased due to repeated exposure. Habituation is linked to stress neurobiology and a reduction in the hypothalamic-pituitary-axis response (13). Pigs are intelligent animals that respond well to operant conditioning techniques used for habituation and counterconditioning to aversive stimuli (3, 12). It is generally recommended that minipigs be habituated to restraint in the sling prior to study to decrease their stress response (12).

Previous studies by Hemsworth et al. (14, 15) have demonstrated that aversive interactions with humans for 30 s 5 days/week or 3 min 3 days/week can contribute to slower growth, higher levels of cortisol, and increased fear toward humans. These effects were observed in pigs handled individually and others in a group subjected to aversive handling (15). Pigs will generalize negative experiences across human handlers, suggesting that aversive techniques used by one handler can induce a fear response to other humans (16, 17). Fear toward humans as a result of previous aversive handling can be overcome through positive handling (17). Pigs can develop positive relationships with humans through positive contact. Positive interactions between animals and humans also are beneficial for handler attitudes and mental health (18).

The goal of this study was to refine handling and restraint techniques for Göttingen minipigs in a research setting by incorporating operant conditioning to improve human-animal interactions and reduce stress during handling and restraint. Another goal of this project was to provide recommendations on best practices for interacting with and restraining pigs for routine study procedures. The procedures outlined in this study occurred within the first 14 d following pig arrival to the research facility. Handling procedures included gentle handling and habituation to the presence of humans, and low-stress placement and restraint in a sling for blood collection. The study aim was to investigate the effect of low stress handling and operant conditioning on pigs' behavioral and physiologic responses to restraint and human interactions.

## Materials and methods

All pig housing and handling protocols were approved by the Institutional Animal Care and Use Committee under the pig colony protocol (999-968G). The facility is accredited by AAALAC, International.

### Animals and housing

Animals were housed at a preclinical safety assessment facility in Mattawan, MI, USA, in May 2021. Forty-four Göttingen minipigs (22 males and 22 females) from Marshall BioResources (North Rose, NY, USA) arrived at the site at ~4 months of age (average weight 8.1 kg). Upon arrival, pigs were examined for clinical and behavioral health. Pig rooms were kept at 16–27°C with 30–70% relative humidity and a 12-h light/dark cycle. Pigs were fed 300 g of Lab Mini-Pig Diet 5081/5K1G (Purina, Lansing, MI, USA) split between 2 daily feedings. Pigs had ad libitum access to water from automatic nipple water systems.

The study took place during a 14-day habituation period prior to pigs being placed on a safety assessment study. Pigs were individually housed due to subsequent study requirements. Pigs were housed in elevated floor pens (Suburban Surgical, Wheeling, IL, USA) with raised floors (1.67 m^2^; 0.3 m above ground) with Tenderfoot^®^ flooring (Minneapolis, MN, USA).

### Treatments

Animal were randomly assigned to control or treatment group *via* a random number generator (random.org). Different experimental groups were housed in separate rooms. The two experimental groups included: Control (C): animals were not provided habituation or operant conditioning outside of normal procedures; Treatment (T): animals received habituation and operant conditioning for 3 min for 3 days/week.

### Training procedure

**Figure 1:**
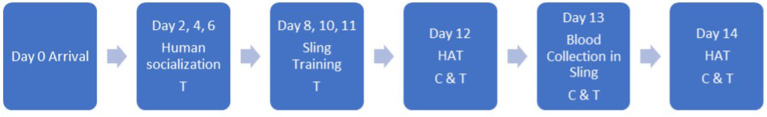
Study timeline including training sessions, human approach tests (HAT), and blood collection (C, control; T, treatment).

Figure 1 presents the study timeline including training sessions, human approach tests (HAT), and blood collection.

The same trainer was used throughout the study and had over 15 years of experience training animals with positive reinforcement techniques.

#### Human socialization

The trainer opened the pen door and started the timer for 3 min. The trainer sat on the pen floor and offered the pig a treat. Small pieces of dried apple were used as the training treat. The trainer offered the pig a target stick (Gear4Pets Retractable Target Stick from Amazon.com) and provided treats if the pig touched the target with its nose. If the pig was target training, this continued for the duration of the 3 min. If the pig was hesitant to touch the target or approach the trainer, the trainer would offer the pig treats by placing them on the pen floor. If the pig would not accept the treats, the trainer would sit passively. After 3 min, the trainer put a treat in the pig's feed bin, stood up and closed the door. Records were kept of which pigs were successfully target trained. On the first day of target training, only 2 pigs touched the target. This improved to 20 pigs by the last day of training. Two pigs never engaged in target training.

#### Sling training

**Figure 2:**
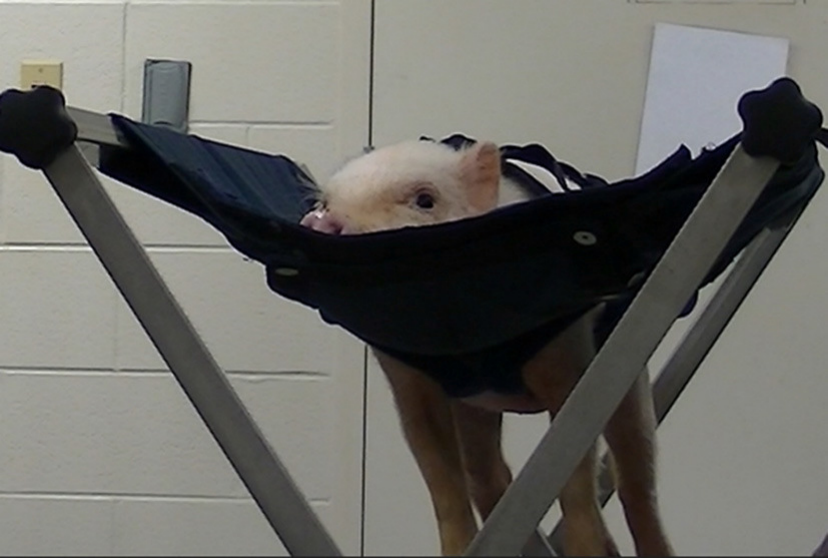
Pig placed in sling during training session.

The trainer opened the pen door and started the timer for 3 min. The trainer sat down on the pen floor and offered the pig a treat. The trainer spent 1 min target training with the pig as outlined above. After 1 min, the trainer picked the pig up (one hand under the thorax and one hand supporting the rump), carried the pig to the sling (Figure 2), and placed the pig in the sling. If a pig displayed stress behaviors (i.e., continuing to run from the trainer, loud squealing and struggling when touched) when the trainer attempted to pick them up, they were not included in sling training. The trainer provided dried apples if the pig was displaying calm behavior in the sling. If all four legs were in their respective holes and the pig was calm, the trainer would buckle the pig into the sling. If the pig was calm, the trainer would also touch the pig along its head and body, each of the four legs, including lightly pinching the legs at the location of the radial and saphenous veins to mimic blood collection. In the event pigs began to panic while in the sling, the trainer would attempt to calm the pig with the treats. If that did not work, the trainer returned the pig back to the pen and continued with target training or sat passively with pig until the end of the training session. When there was 30 s remaining, the trainer removed the pig from the sling and returned it to its pen. With the remaining time, the trainer continued target training with the pig. After 3 min, the trainer placed a food reward in the feed bin, stood up and closed the pen door. One pig was omitted from sling training on day 10 due to stress behavior when being picked up. A total of 3 pigs were removed early from the sling due to stress behavior.

### Behavior measures

#### Human approach test

The human approach test was conducted on days 12 and 14. The test was conducted at the pigs' home pen. A novel human approached the pen, opened the door, and stood at the pen front facing the pig, with eyes averted. The test duration was 5 min. The human approach test was video recorded and scored by a single trained observer blinded to study treatments and animal sex. Intraobserver reliability was assessed by rescoring 7 videos and reached 100% agreement for 6 of 7 videos and 86.7% agreement for 1 of 7. Pigs were scored on latency to touch the human observer (s), and duration of time touching the observer (s).

#### Sling restraint test

The sling restraint test was conducted on day 13 of the study while the pigs were restrained in the hammock sling (Lomir Sling for Ellegaard Göttingen Minipig, Malone, NY, USA) for blood collection. Pigs were video recorded during blood collection. The videos were later scored for pig behavior while in the sling using the ethogram in Table 1 for up to 1 min. Behavior in the sling was scored by a single trained observer blinded to the study treatment and sex of the pigs. Intraobserver reliability was assessed by rescoring 4 videos. Percentage of agreement ranged from 88.4 to 97.8%. Duration of time (s) in sling for blood collection was also recorded.

**Table 1:** Sling restraint test ethogram.

**Behavior**	**Description**	**Sampling method**
Struggle	Pig is moving body and legs including side to side body movements, kicking legs, picking legs up out of the sling.	Duration of time struggling (s)
Vocalizations	Pig makes a squealing sound (grunts were not included as they could not be distinguished from background noises of the other pigs in the room).	Duration of vocalizations (s)

#### Behavior recording and scoring

Behavior in the home pen, including the human approach tests, were recorded using Reolink (RCL-410-5mp; Wilmington, New Castle, Delaware, USA) cameras affixed to the pens. Videos were observed and scored using Noldus Observer XT 15 (version 15.0.1200; Leesburg, VA, USA). Training sessions and behavior in the sling were recorded using camcorders (JVC GZ-E200BU; Walnut, CA, USA).

### Physiologic measures

#### Blood collection and cortisol analysis

Pigs were placed into a hammock sling for blood collection from the radial vein. Prior to blood collection, technicians applied lidocaine cream (EMLA Topical Cream, Actavis, Parsippany, NJ, USA) to the two front legs around the location of radial vein collection. Technicians used ~½ tsp and rubbed it onto the pigs' legs. The lidocaine was applied at least 5 min prior to blood collection, and not longer than 20 min. Technicians collected ~2 mL of blood into barrier-free, additive-free silica/PET serum vacutainer collection tubes. The clotted blood was centrifuged at 1,300 × rcf for 10 min under ambient conditions to separate the serum. Serum samples were harvested and transferred into microtubes and subsequently stored frozen at −60°C until thawed for determination of cortisol level by automated chemiluminescent immunoassay using the Unicel DxI 600 Access Immunoassay System (Beckman Coulter, Inc., Brea, CA, USA).

#### Body weight

Pigs were weighed upon arrival and at day 15.

### Statistical analyses

All analyses were conducted using R Studio (2020; Vienna Austria. URL https://R-project.org/). Data were assessed for normality by visual inspection of the histogram and quantile-quantile plot and using the Shapiro-Wilk test. Human approach test variables latency to touch and duration of time touching were square root transformed. Sling behavior variables struggling and vocalization were also square root transformed.

Relationships between treatment groups and the response variables of interest (latency to approach novel human, duration of time in contact with novel human, duration of time spent struggling in the sling, vocalizations in the sling, serum cortisol levels, and weight) were analyzed using separately fitted linear models with treatment and sex as fixed effects. For bodyweight, animal number was included as a random effect to account for repeated measures.

For the human approach tests (HAT), a linear mixed model was fitted initially with latency to approach as the response variable, timing of HAT (pre or post), treatment, and sex as fixed effects, and animal number as a random effect. The results revealed significant effects of treatment and the interaction between treatment and time of HAT test (pre or post) on latency to approach. To investigate these results further, each HAT was analyzed separated with linear models with latency to approach as the response variable, and treatment and sex as fixed effects.

## Results

### Human approach tests

**Figure 3:**
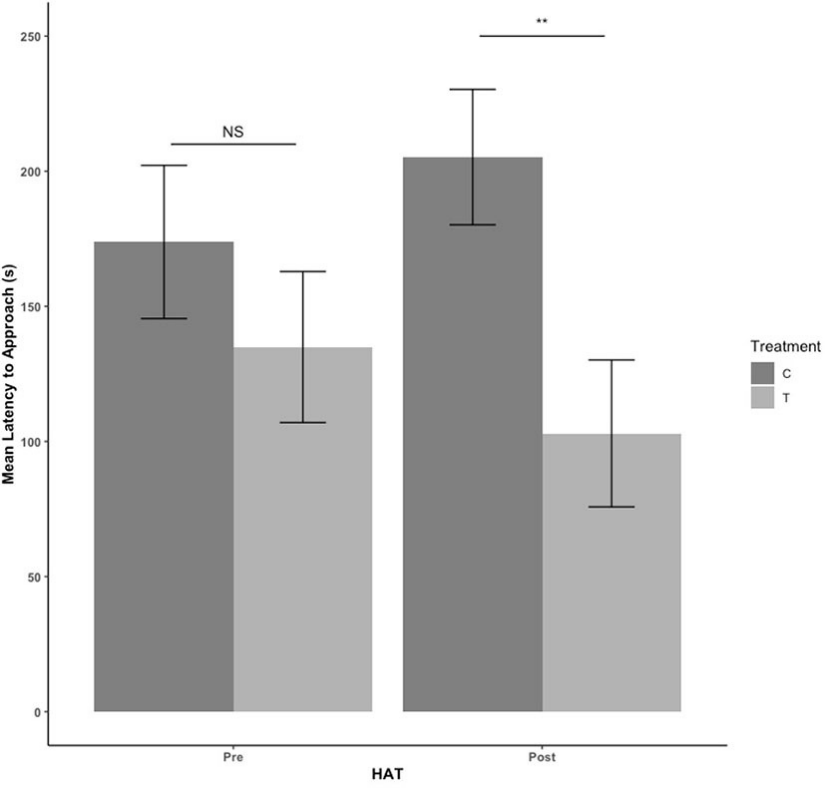
The mean latency to approach during the novel human approach test. Tests were conducted the day before blood collection (pre) and the day after (post). Control (C) pigs received no habituation or training. Treatment (T) pigs received 3 min of habituation and training 3 days per week. NS, *P* > 0.05; **P ≤ 0.01.

**Figure 4:**
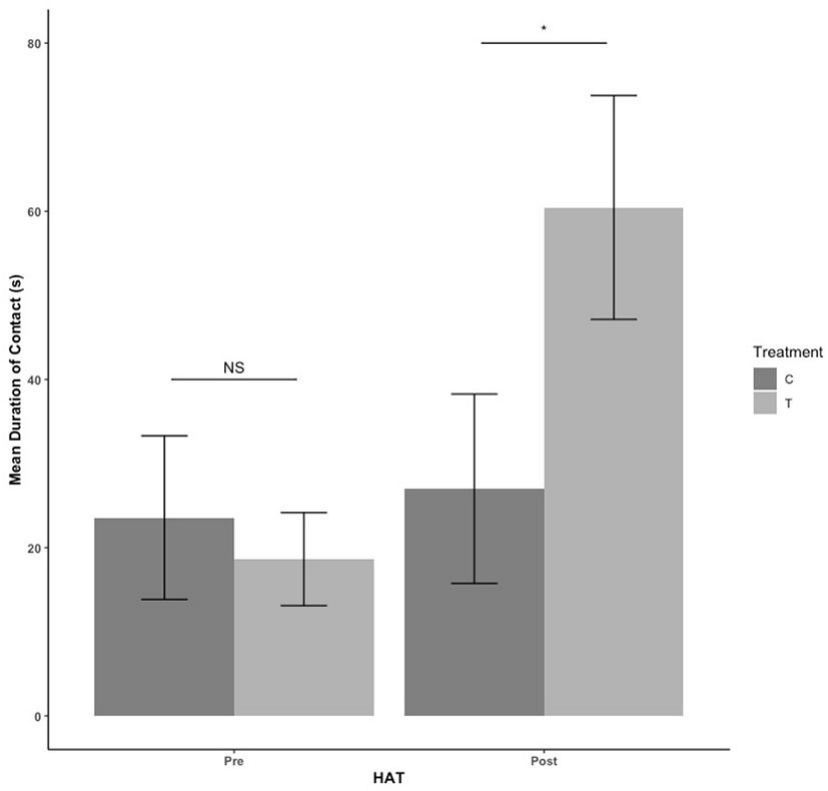
The mean duration of contact with the human during the novel human approach test. Tests were conducted the day before blood collection (pre) and the day after (post). Control (C) pigs received no habituation or training. Treatment (T) pigs received 3 min of habituation and training 3 days per week (Figure 2). NS, *P* > 0.05; *P < 0.05.

There were no treatment or sex effects in the pre-blood collection HAT (*P* > 0.05). There was an effect of treatment for the post-blood collection HAT, with T pigs having a shorter latency to approach [*F*_(1,41)_= 9.684, estimate: −5.352, se: 1.72, *P* = 0.003] as seen in Figure 3. For duration of time spent in contact with the human during HAT, there was no treatment effect pre-blood collection (*P* = 339). After blood collection, T pigs spent more time in contact with the human compared with C pigs [*F*_(1,41)_ = 4.560, estimate: 3.091, se: 1.448, *P* = 0.039] in Figure 4.

### Sling restraint test

There was no difference in sling behavior between C and T pigs, including struggle behavior during blood collection (*P* = 0.418), vocalizations (*P* = 0.060), and total time to collect blood (*P* = 0.282).

### Serum cortisol levels

**Figure 5:**
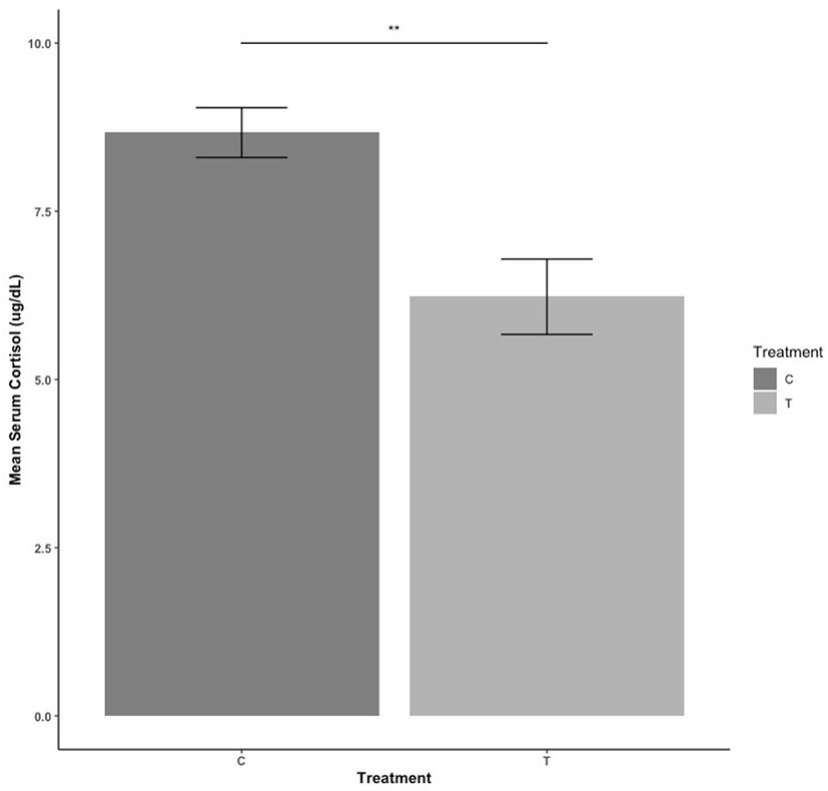
Serum cortisol levels during blood collection compared between control (C) pigs who received no habituation or training and treatment (T) pigs who received 3 min of habituation and training 3 days per week. NS, *P* > 0.05; **P ≤ 0.01.

Serum cortisol levels for treatment animals were lower than control animals [*F*_(1,30)_ = 14.109, estimate = −2.360, se = 0.657; *P* = 0.001] (Figure 5). There were no sex differences (*P* > 0.05).

### Body weight

The average body weight at the start of the study was 8.11 kg (7.99 kg for males; 8.21 for females). The average weight at the end of the study was 10.23 kg (10.23 kg for males; 10.24 for females). There were no treatment (*P* = 0.673) or sex differences (*P* = 0.647).

## Discussion

The aims of this study were to refine handling and restraint techniques for Göttingen minipigs during the first 2 weeks on site and provide recommendations for habituation procedures. The results of this study suggest that using habituation and operant conditioning techniques for 3 min three times/week in the first 2 weeks after arrival at a facility is sufficient to reduce stress during restraint and promote positive-human animal interactions. Pigs that received training had lower serum cortisol levels during blood collection and were more willing to interact with humans the day after blood collection compared with pigs who received no training.

Pigs who received training were more likely to approach a novel human the day after blood collection even though there was no difference in response to a novel human prior to blood collection, suggesting improved association with humans after a stressful event. Control pigs received basic husbandry including feeding and cleaning pens. The results also suggest that without the influence of aversive procedures, pigs have similar responses to novel humans but their response after an aversive event is more indicative of their association with humans. Previous studies by Hemsworth et al. also demonstrated that it takes minimal interactions between pigs and humans to affect the valence of the relationship (14–16). The same can be true of positive interactions—that it takes minimal effort on a consistent basis to build a positive relationship and decrease stress (18, 19). Tactile contact, such as stroking and scratching, can promote positive human-pig relationships, resulting in anticipatory behavior from pigs for human tactile contact and pigs actively seeking out human contact (18). Positive interactions with pigs often include the use of their flight zone and point of balance to non-invasively move them in the desired direction, as well as using slow movement and a quiet, calm voice to speak to pigs (20). Pigs that are handled in a positive manner, such as gentle stroking, have lower serum glucocorticoid concentrations compared to pigs that are handled with aversive techniques, such as electric prods (14). Other negative interactions with pigs may include invasive handling techniques such as slapping, kicking, shouting, and shoving (20). The benefits of positive human-animal interactions have been reported in other species, including laying hens, in which positive human interactions (15 min of additional human contact 5 days/wk) promoted less fear toward humans, greater cell-mediated immunity, and increased egg production (21). Target training is an easy behavior to teach pigs due to their natural curiosity to explore with their noses, and the target can be used to encourage pigs to move cooperatively to a desired location. Pigs can be trained to follow a target to walk onto a scale or platform, removing the need to carry minipigs for procedures.

The training techniques resulted in reduced serum cortisol levels of pigs while they were in the hammock sling for blood collection; however, there was no difference in behavior in the sling, either amount of struggling or vocalizations, suggesting these techniques were not sufficient to promote calmer behavior. Restraint is a common procedure for research pigs but may cause stress and altered physiological states that are disruptive to collecting good data from these study subjects. In a study by Stephens and Rader (4) pigs that were restrained (lifted in a harness so that the front legs were off the ground) had increased heart rate and blood pressure and decreased renal blood flow. Escribano et al. (7) reported differences in salivary proteins between pigs restrained by a nose snare and control pigs that were not restrained. Cornulin, heat shock protein 27, and lactate dehydrogenase increased with acute stress while immunoglobulin J chain decreased (7). Parrott and Lloyd (5) found that pigs restrained with the nose snare showed increased body temperature and serum cortisol levels, despite administration of indomethacin (5). Epinephrine, norepinephrine, dopamine, and serum cortisol all increased within 5 min of restraint and continued to increase the longer they were restrained (6).

For minipigs, manual restraint is commonly used while larger research pigs may be restrained using nose snares. Both of these methods put the animal and handler at risk for injury and cause stress to the animals. Pigs are intelligent animals that respond well to training techniques; however, the use of positive reinforcement and target training to encourage voluntarily cooperation during husbandry and research procedures is not common (22, 23). Yucatan hairless pigs have been trained to cooperate with daily non-invasive skin analysis (24). There were no effects to the data collected compared to data collected from anesthetized pigs (25). Training staff to use these techniques correctly and consistently and providing the time and resources to implement training programs with pigs would improve welfare for pigs and research personnel.

The limitations in this study include the inability to include additional training groups to better explore increased training time after arrival to promote calm behavior in the sling. In the study a more refined blood collection technique (radial vein) was used that had not been used previously at the site. Technicians were trained on the technique but were not as experienced with this method. Peripheral blood collection and microsampling are refinements being promoted within research environments to reduce stress on animals. Another limitation was the time allotted to complete the habituation. Ideally, animals should be given at least 1 week to acclimate to the facility upon arrival to allow them to recover from transportation stress (22, 23, 26) and they may require up to 2 weeks to habituate to personnel (25). Future studies should investigate a period of 4 weeks to prepare pigs for study, including 1 week to acclimate, and 3 weeks for habituation and training for research procedures. A final limitation with the study was that pigs were individually housed due to study constraints. Pigs are social animals such that the effects of training and response toward humans likely would be affected by the presence of conspecifics. Future studies should investigate the influence of conspecifics on training and the effectiveness of group training with pigs.

In conclusion, use of habituation and operant conditioning techniques is an important refinement for research pigs and promotes reduced stress during restraint for blood collection as well as improving human-animal interactions following procedures. Implementing these techniques upon animal arrival can be effective in as little as 3 min three times/week, although more time may be needed to see improvements in sling restraint behavior.

## Data availability statement

The raw data supporting the conclusions of this article will be made available by the authors, without undue reservation.

## Ethics statement

The animal study was reviewed and approved by Charles River Mattawan Institutional Animal Care and Use Committee, Mattawan, MI, USA.

## Author contributions

CO'M and PT: study design. CO'M, RH, and HT: data collection. CO'M: data analyses and writing. CO'M, RH, HT, and PT: MS review. PT: editing and guidance. All authors contributed to the article and approved the submitted version.

## Funding

All funding was provided by Charles River Laboratories.

## Conflict of interest

All authors were employed by company Charles River Laboratories. The remaining authors declare that the research was conducted in the absence of any commercial or financial relationships that could be construed as a potential conflict of interest. The authors declare that this study received funding from Charles River. The funder was not involved in the study design, collection, analysis, interpretation of data, the writing of this article or the decision to submit it for publication.

## Publisher's note

All claims expressed in this article are solely those of the authors and do not necessarily represent those of their affiliated organizations, or those of the publisher, the editors and the reviewers. Any product that may be evaluated in this article, or claim that may be made by its manufacturer, is not guaranteed or endorsed by the publisher.
